# Current Incentives for Scientists Lead to Underpowered Studies with Erroneous Conclusions

**DOI:** 10.1371/journal.pbio.2000995

**Published:** 2016-11-10

**Authors:** Andrew D. Higginson, Marcus R. Munafò

**Affiliations:** 1 Centre for Research in Animal Behaviour, College of Life and Environmental Sciences, University of Exeter, Exeter, United Kingdom; 2 MRC Integrative Epidemiology Unit (IEU) at the University of Bristol, Bristol, United Kingdom; 3 UK Centre for Tobacco and Alcohol Studies, School of Experimental Psychology, University of Bristol, Bristol, United Kingdom

## Abstract

We can regard the wider incentive structures that operate across science, such as the priority given to novel findings, as an ecosystem within which scientists strive to maximise their fitness (i.e., publication record and career success). Here, we develop an optimality model that predicts the most rational research strategy, in terms of the proportion of research effort spent on seeking novel results rather than on confirmatory studies, and the amount of research effort per exploratory study. We show that, for parameter values derived from the scientific literature, researchers acting to maximise their fitness should spend most of their effort seeking novel results and conduct small studies that have only 10%–40% statistical power. As a result, half of the studies they publish will report erroneous conclusions. Current incentive structures are in conflict with maximising the scientific value of research; we suggest ways that the scientific ecosystem could be improved.

The career progression of researchers is strongly influenced by their publication record [1], but there is growing evidence that many published studies across a number of disciplines may be underpowered and report erroneous conclusions [2–4]. In 2005, Ioannidis argued that most published research is false [5] and that this stems in part from a reliance on null hypothesis significance testing and in particular from a dichotomous interpretation of *p*-values as “significant” or “nonsignificant,” whereas the positive predictive value (PPV) of a study (i.e., the poststudy probability that the finding is correct) is a better measure of the scientific value of a study. In particular, the prestudy odds (*R*) that a hypothesis is correct are rarely considered when interpreting the results of individual studies, yet this can have a dramatic impact on the PPV. Exploratory studies (i.e., those with low *R*) are much less likely to be true than confirmatory studies (i.e., those with high *R*) even if the *p-*value generated is the same, but arguably, current incentive studies reward novel (i.e., exploratory) findings over replication (i.e., confirmatory) studies.

Scientists are trained to be objective and to pursue the discovery of knowledge, through both exploratory work that generates novel lines of enquiry and confirmatory work that assesses the robustness of previous novel findings. However, scientists are also human and work within incentive structures that may shape their behaviours, consciously or unconsciously. For example, publication in a journal with a high (Thomson-Reuters) Impact Factor can accelerate career advancement [1], enhancing prestige and both personal and grant income. There also appears to be an increasing focus on novel findings: since the 1980s, there has been a disproportionate increase in studies that include “novel” in their title [6–9]. At the same time, only a small number of key publications may count towards career advancement: recruitment panels and research assessment exercises, such as the United Kingdom Research Excellence Framework (REF) [9] and the Australian Excellence in Research exercise [10], often require researchers to submit for assessment a small number (currently four in the REF) of their “best” outputs. We can regard these incentive structures as an ecosystem within which scientists strive to maximise their fitness (i.e., publication record) and therefore might expect that individual scientists would strategically adapt—consciously or unconsciously—to these pressures, adjusting their research strategy to boost their career success. Understanding research ecosystems is critical if we are to align scientific value and career benefits, in order to maximise the efficiency of scientific research.

Theoretical models of adaptive behaviour are common in evolutionary biology: natural selection should find, or an animal should choose, the behavioural strategy (a set of context-dependent choices) that maximises naturally selected "fitness," or "reproductive value" [11]. Possible strategies available to natural selection or in decision making can be thought of as lying in multidimensional space with a peak at maximum fitness. We can regard the wider incentive structures that operate across science as the characteristics of an ecosystem, within which scientists strive to maximise their “fitness” by optimising their behaviour and, by extension, their research strategy. We were interested in whether the optimal research strategy for individual scientists aligns with the optimal conditions for the advancement of knowledge.

We used optimality theory [12] to predict the rational strategy of a scientist possessing finite resources who seeks to maximise the career value of his or her publications. The model is described in brief in Box 1. Full details of all methods used are provided in S1 Text, and the Matlab code used to complete the analyses is provided in S2 Text. We considered that researchers must choose how to divide their resources between exploratory studies that seek to identify new phenomena and confirmatory studies that attempt to verify previous findings and that they must decide the amount of resources to invest per study. We characterised the possible strategies as lying in a two-dimensional “fitness” landscape (Fig 1) in which the two dimensions are (1) the proportion of research effort spent on exploratory studies that seek novel results (*θ*) and (2) the amount of research effort (e.g., sample size) per exploratory study (*S*_*E*_). For instance, we might assume that collecting one data point has a fixed monetary cost or takes a certain amount of time to collect. For simplicity, we assume that exploratory studies are published only if they obtain a statistically significant result (i.e., *p* < 0.05) and that confirmatory studies are large (i.e., have a large sample size), have high power, and therefore have a high probability of being accepted for publication even if they obtain a nonsignificant result. We also assume that the peer review process means that the likelihood of acceptance for publication increases with sample size, since larger studies are generally considered informative and authoritative, irrespective of whether the result is statistically significant or not.

**Fig 1:**
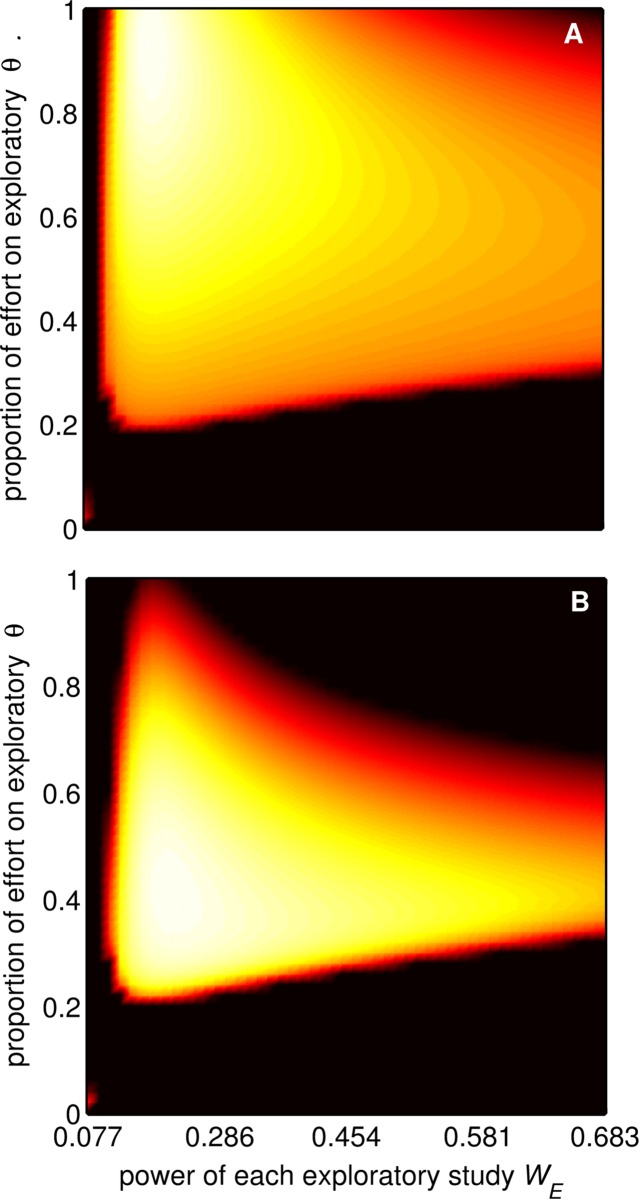
Fitness landscape for an individual researcher. An individual researcher is able to choose the parameters *θ* (*y*-axis) and *S*_*E*_; the *x*-axis shows the resultant power of exploratory studies, *W*_*E*_. White indicates high fitness, black low fitness. For small values of *S*_*E*_, few papers are accepted, while for high values of *S*_*E*_, few studies are carried out. For low values of *θ*, few novel studies are carried out. (A) γ = 0.09, ϕ = 0.9. The optimal strategy that maximises individual fitness is therefore to carry out many small exploratory studies with a power of around 15%. (B) γ = 0.055, ϕ = 0.55. A mixture of exploratory and confirmatory work should be carried out with slightly higher power (20%).

**Box 1. Modelling the Rational Strategy of a Scientist**

The researcher optimises the payoff that results from deciding the proportion of total sampling to spend on exploratory studies (*θ*) and the sample size of each exploratory study (*S*_*E*_). *S*_*E*_ determines the statistical power of each study, namely the probability of detecting an effect (*W*_*E*_), which in turn controls the probability of Type II errors (1 − *W*_*E*_). The probability of a Type I error (α) is the critical *p-*value within a null hypothesis significance testing framework; we assume α = 0.05. Power depends on the population variance (σ^2^) and the effect size for exploratory or confirmatory studies (*r*_*E*,_ *r*_*C*_). The values we use are in the middle of the range of effect sizes observed in meta-analyses across a number of biomedical research domains (range r ~ 0.15 to 0.50) [13]. All studies that find significant results are published, and the high statistical power of confirmatory studies means that the results are informative regardless of statistical significance, so nonsignificant confirmatory studies are published with probability ψ, subject to an independent effect of sample size on the likelihood of acceptance by a journal editor (before consideration of the effects themselves) according to the function:

$$A=1-\frac{m}{S_{i}},$$ (B1)

where *m* is a positive constant (see S1 Fig).

The number of publications from exploratory studies (*N*_*E*_) is the product of the total effort put into exploratory studies divided by the sampling effort of each study, the probability of acceptance given the sample size *A*, and the probability of getting a statistically significant result:

$$N_{E}=\frac{\theta T}{k+2S_{E}}A\left[W_{E}f_{E}+\alpha (1-f_{E})\right],$$ (B2)

where *f*_*E*_ is the probability that an effect is real, *T* is the total number of samples that can be collected (i.e., total resources), and *k* is the setup cost for any study. The first term in the squared brackets is the probability of a true-positive result, while the second is the probability of a false-positive result. Since confirmatory studies will build on the findings of exploratory studies, the probability that a confirmatory study is looking at a real effect (*f*_*C*_) is equal to the probability that a published exploratory study is correct *(P_F,E_)*. The number of confirmatory studies that are published (*N*_*C*_) is the sampling effort put into all confirmatory studies divided by the sampling effort of each study multiplied by the probability they are accepted:

$$N_{C}=\frac{(1-\theta )T}{k+2S_{C}}\left[\left(1-P_{F,E}\right)W_{C}+P_{F,E}\alpha +\psi f_{C}(1-\alpha )+\psi \left(1-P_{F,E}\right)\left(1-W_{C}\right)\right].$$ (B3)

The terms in the squared brackets are, respectively, the probability of true positive, false positive, true negative, and false negative.

We assume that the number of confirmatory studies per exploratory study is limited to ρ (ρ = 10), by calculating the number of valuable confirmatory studies ${\hat{N}}_{C}$ (see S1 Text).

The total fitness (*V*_*R*_) of the researcher is assumed to depend on total number of publications with diminishing returns, with an additional bonus for exploratory studies. One implementation of this is as follows:

$$V_{R}=\gamma N_{E}+1-e^{-\varphi \left(N_{E}+{\hat{N}}_{C}\right)}.$$ (B4)

In Eq (B4), ϕ controls how quickly the value of the total number of publications diminishes, and γ controls the extra weighting given to published exploratory studies. The dependence of equation (B4) on the number of published exploratory and confirmatory studies is shown in S2 Fig for representative values of γ and ϕ.

Given these assumptions, we identified the optimal research strategy for an individual scientist, which is the combination of *θ* and *N*_*E*_ for which the career value of publications (*V*_*R*_) is maximised (i.e., the location of the peak in the fitness landscape, Fig 1). See S2 Text for the Matlab code.

A reasonable function describing the scientific value of research (*V*_*S*_) is the product of (1) the number of published exploratory studies, (2) the number of published confirmatory studies, and (3) the proportion of published studies that are correct: *V_S_* = *N_C_N_E_*(1−*P_F_*). This reflects our assumptions that novel findings (from exploratory work) and confirmatory work are equally important for the advancement of knowledge, provided they arrive at correct conclusions, and that an absence of either of them would be very bad for science. That is, we assume that a balance of exploratory and confirmatory work is ideal, so that for any number of publications the scientific value is maximised when half are exploratory and half are confirmatory (see S3A Fig). We also considered other reasonable functions, and these provided the same conclusions (see S3 Fig and S5–S9 Figs). Baseline parameter values are given in S1 Table.

The results (Fig 2A) of our model (Box 1) indicate that more exploratory work will be carried out if (1) more weight is given to novel findings (γ, shown on the *x*-axis), (2) real effects are more common (*x*_*E*_, compare dotted and solid lines), and (3) the typical effect size is larger (*r*_*C*_, *r*_*E*_, compare dashed and solid lines). There is an optimal sample size (*S*_E_*) that maximises the number of published novel findings per unit of resource spent on exploratory studies. *S*_E_* decreases as θ increases (Fig 2B), because it becomes more important to avoid committing false positives as they will reduce the number of confirmatory studies that find a significant result. The total number of publications declines as the weight given to novel findings (γ) increases (Fig 2C). In part, this is because most exploratory studies are not published, as they have low statistical power and therefore often do not obtain statistically significant results. However, the proportion of confirmatory studies also declines (Fig 2D) because of the greatly increased exploratory effort. The optimal statistical power is low (Fig 2E), especially if the typical effect size is small, since it is better from an individual career perspective to run many exploratory studies (and for a high proportion of statistically significant findings to be Type I errors [2]) than to run a smaller number of well-powered studies (see S4 Fig for an intuitive illustration). As the weight given to novel findings increases, and so the investment in exploratory studies increases, the proportion of papers that draw erroneous conclusions increases to over 50% (Fig 2F). The proportion of false positive studies at optimal behaviour is similar to the proportion incorrect, since false negatives are rare for confirmatory studies because they have high statistical power and false negative exploratory studies tend to remain unpublished.

**Fig 2:**
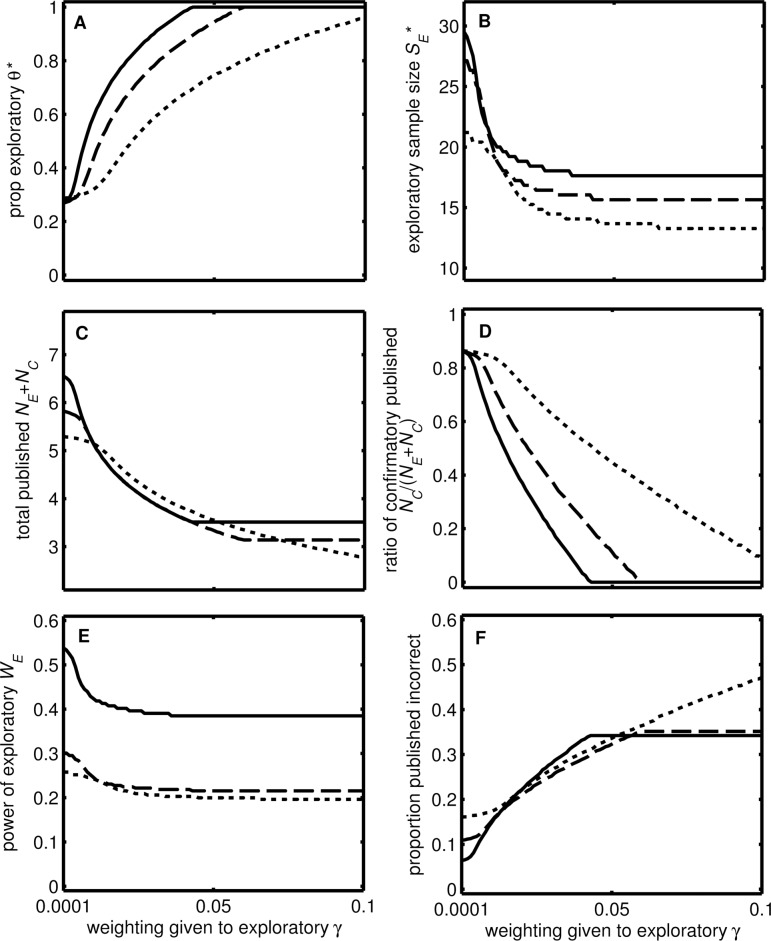
Effect of varying the weighting given to published exploratory studies (γ). Parameter γ reflects the relative importance of published exploratory studies. The lines show predictions for two values of the probability that an effect is real (*f*_*E*_) and two values of the effect sizes *r*_*C*_ and *r*_*E*_ (solid: *f*_*E*_ = 0.2, *r*_*C*_ *= r*_*E*_ = 0.21; dotted: *f*_*E*_ = 0.3, *r*_*C*_ *= r*_*E*_ = 0.21; dashed: *f*_*E*_ = 0.2, *r*_*C*_ *= r*_*E*_ = 0.32). The panels show (A) the optimal proportion of total sampling to spend on exploratory studies θ*, (B) the optimal sample size of exploratory studies *S*_E_*, (C) the resultant total number of published studies *N*_*E*_ + *N*_*C*_, (D) the proportion of published studies that are confirmatory *N*_*C*_ / (*N*_*E*_ + *N*_*C*_), (E) the statistical power of exploratory studies *W*_*E*_, and (F) the proportion of published studies that draw incorrect conclusions (*P*_*F*_). Other values: *S*_*C*_ = 120, *T* = 2,000, *k* = 20, α = 0.05, σ^2^ = 1, *m* = 3, and ϕ = 0.8. The chosen values for *r*_*C*_ *= r*_*E*_ reflect data reported by Richard and colleagues [14], where a correlation coefficient mode of 0.09 and a mean of 0.21 were observed. These values are in the middle of the range of effect sizes observed in meta-analyses across a number of biomedical research domains (range r ~ 0.15 to 0.50) [13].

We next used our model to predict how characteristics of the current scientific ecosystem, such as incentives to publish novel, exciting results, influence the total scientific value of research *V*_*S*_ (see Box 1). Current incentive structures (e.g., recruitment processes and research assessment exercises) place substantial weight on findings published in journals with a high Impact Factor and may consider only the “best” few publications of any individual. These conditions correspond to a situation with a strong weighting given to novel findings (large γ) and quickly diminishing value of additional publications (high ϕ). Our model shows that the scientific value of research (*V*_*S*_) is not maximised at these values (top right of Fig 3A) when scientists are behaving rationally to maximise their own success within this ecosystem. If a small number of novel findings counts heavily towards career progression, this encourages scientists to focus almost all of their research effort on underpowered exploratory work. Furthermore, they should carry out lots of underpowered small studies to maximise their number of publications, even though this means around half will be false positives (Fig 2E and S4 Fig).

**Fig 3:**
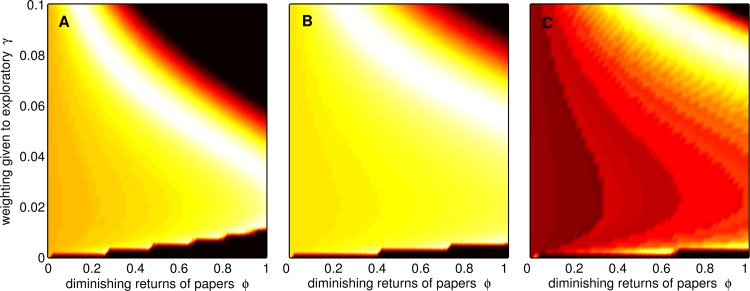
Effect of γ and ϕ on a hypothetical measure of the total scientific value of research (*V*_*S*_). The figure shows the product of the number of published confirmatory studies, the number of published exploratory studies, and the proportion of published studies that are correct (red = high, blue = low). This measure is calculated for when all researchers are following the rational strategy given the values of γ and ϕ. The current emphasis on a small number of publications that report novel findings is characterised by high γ and high ϕ (top right). To improve scientific output according to this measure, we could reduce ϕ (i.e., make more published studies count for researchers’ careers) or reduce γ (i.e., reduce weighting of published exploratory studies). Interestingly, the ridge is flat, so any point along it has equal fitness. Therefore, a pragmatic compromise would be to reduce both γ and ϕ by a lesser amount. The panels show the *V*_*T*_ for two values of the dependence of acceptance on sample size *m* and the Type I error rate α: (A) α = 0.05, *m* = 3, colour range: 2.0–3.18; (B) α = 0.05, *m* = 6, colour range: 2.0–2.82; (C) α = 0.03, *m* = 6, colour range: 2.0–2.065. Other values: *S*_*C*_ = 120, *T* = 2000, *k* = 20, *f*_*E*_ = 0.2, *r*_*C*_ *= r*_*E*_ = 0.21, and σ^2^ = 1.

Critically, our model suggests ways in which incentive structures could be redesigned so that the optimal strategy for individual scientists aligns with the optimal conditions for the advancement of knowledge. A small reduction in both the weight given to novel findings (γ) and how quickly the value of the total number of publications diminishes (ϕ) would shift individual incentives away from a dominant focus on exploratory work, meaning that more confirmatory work is carried out, thereby increasing the total scientific value of research (Fig 3A). Sensitivity analyses (S5–S9 Figs) indicate that a reduction in both ϕ and γ increases *V*_*S*_ for all reasonable values of the other parameters (i.e., setup cost, *k*; probability effect is real, *f*_*E*_; effect size in exploratory studies, *r*_*E*_; effect size in confirmatory studies, *r*_*C*_; standard deviation of the data, σ; and proportion of nonsignificant confirmatory studies published, ψ). This suggests that the optimal strategy for individual scientists and the optimal conditions for the advancement of knowledge do not currently align even if our estimates of parameter values are incorrect. The equation for *V*_*S*_ assumes that there is a place for both exploratory and confirmatory research and that correct findings are more valuable than incorrect findings. How these elements should be weighted is obviously an important question, but our results indicate that our conclusions are unchanged for various possible functions for *V*_*S*_ (see S5–S9 Figs).

Our metric reflecting the scientific value of research (*V*_*S*_) is related to both the dependence of acceptance on sample size (*m*) and the Type I error rate (α) (Fig 4). As the dependence of acceptance on sample size increases (i.e., journal editors are more stringent), so does statistical power, meaning that the proportion of studies that are correct increases, tending to 100% (Fig 4A). More confirmatory studies are carried out, so the number of studies that get published increases (Fig 4C). However, when the sample size required for publication is very large, the number of exploratory studies approaches zero, so that the total scientific value of research declines (Fig 4E). This analysis predicts that the value of *m* that maximises the scientific value of research is quite high, meaning that journals should be more stringent about required statistical power and sample size. Increasing *m* alters the position of the ridge in (γ, ϕ) space, such that total scientific value of research is greatest at larger values of γ and ϕ (Fig 3B).

**Fig 4:**
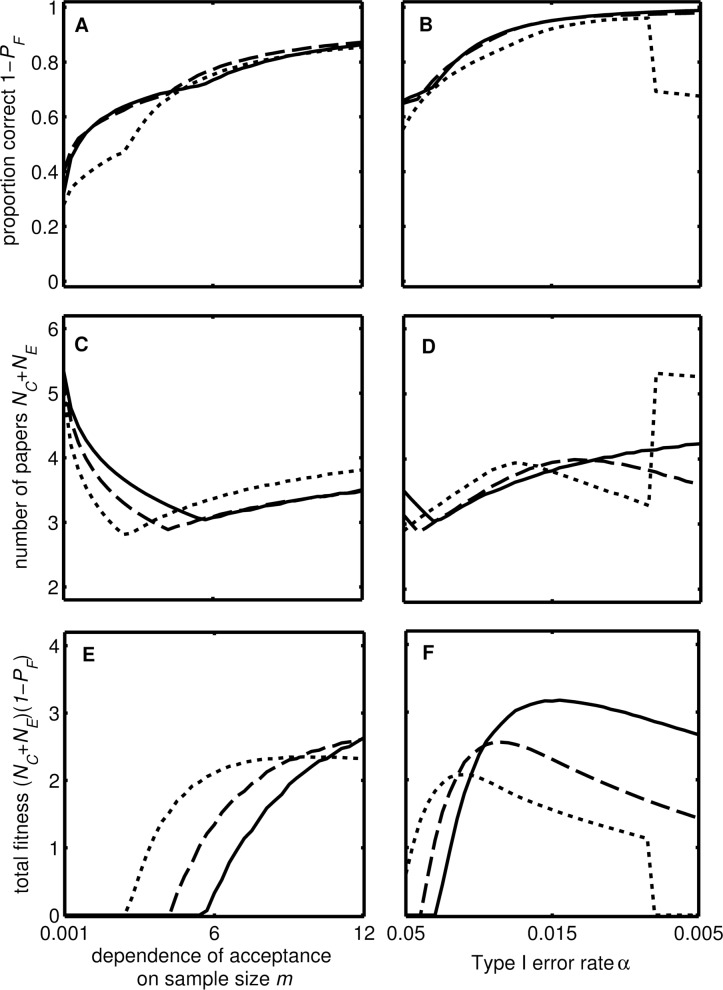
Effect of editorial stringency on total scientific output for current incentive structures. The figure shows the proportion of published findings that are correct 1-*P*_*F*_ (A, B), the total number of published studies *N*_*C*_ + *N*_*C*_ (C, D), and the total scientific value of research *V*_*T*_ (E, F). We varied the following parameters: the probability of a Type I error α (A, C, E), and the dependence of acceptance on sample size *m* (B, D, F). The lines show predictions for two values of the probability that an effect is real (*f*_*E*_) and two values of the effect size (solid: *f*_*E*_ = 0.2, *r*_*C*_ *= r*_*E*_ = 0.21; dotted: *f*_*E*_ = 0.3, *r*_*C*_ *= r*_*E*_ = 0.21; and dashed: *f*_*E*_ = 0.2, *r*_*C*_ *= r*_*E*_ = 0.32). Other values: *N*_*C*_ = 120, *T* = 2,000, *k* = 20, α = 0.05, σ^2^ = 1, *m* = 3, ϕ = 0.9, and γ = 0.09. The steps occur where there is discontinuity in the effect of α on *S*_E_*.

Our model indicates that, at conventional levels of statistical significance (i.e., α = 0.05, Fig 4B), only around 50% of published findings are likely to be correct, close to the ~40% observed by the Open Science Collaboration [4] and the pharmaceutical industry [15]. With increasing statistical stringency (i.e., lower α), the proportion of published findings that are correct increases (Fig 4B) because more confirmatory studies are carried out and exploratory studies are more powerful because it is optimal to have a larger sample size in order to increase the chance of detecting an effect. This, along with an increase in the number of published studies, means that *V*_*S*_ increases (Fig 4D). However, at small values of α almost all published papers are confirmatory, and a single novel result would greatly increase *V*_*R*_, so *S*_*E*_ increases as α decreases to maintain the chance of a statistically significant result. However, at very high levels of statistical stringency (i.e., very low α) fewer novel findings are published and so the total scientific value of research declines. In a similar way to the result for *m* above, this analysis suggests that the total scientific value of research would be greater given a smaller Type I error rate (α) than is currently conventional (Fig 4F). Decreasing α would mean that higher values of γ and ϕ would maximise the total scientific value of research (Fig 3C)—in other words, the research strategy encouraged by current incentives (top right of Fig 3C) would be closer to that which maximises the scientific value of research. The problem with this solution is that the overall value of the science might be reduced (the maximum *V*_*S*_ is smaller in Fig 3C than in Fig 3A).

Current incentive structures in science, combined with existing conventions such as a significance level of 5%, encourage rational scientists to adopt a research strategy that is to the detriment of the advancement of scientific knowledge. Given finite resources, the importance placed on novel findings, and the emphasis on a relatively small number of publications, scientists wishing to accelerate their career progression should conduct a large number of exploratory studies, each of which will have low statistical power. Since the conclusions of underpowered studies are highly likely to be erroneous [2], this means that most published findings are likely to be false [5]. The results of our model support this conclusion. Indeed, given evidence that with sufficient analytical flexibility (known as *p*-hacking) almost any dataset can produce a statistically significant (and therefore publishable) finding [16], our results are likely to be conservative. There is therefore evidence from both simulations and empirical studies that current research practices may not be optimal for the advancement of knowledge, at least in the biomedical sciences.

Ioannidis [5] concluded—on the basis of simulations of the impact of varying types of bias—that most published research findings are false. Button and colleagues [2] showed that the average statistical power of studies in neuroscience is likely to be very low, and there is evidence that this problem exists across different domains of biomedical science [3]. Recently, the Open Science Collaboration [4] reported that of 100 psychology studies selected from leading journals, only a minority of findings (approximately 40%) could be replicated. Similar results have been obtained by the pharmaceutical industry attempting to reproduce “landmark” findings from the published academic literature [15]. A survey of early career researchers indicated that “survival mentoring” (i.e., guidance on how to survive in the profession) is associated with increased odds of questionable behaviour in methods (e.g., withholding details of methodology or results), use of funds (e.g., use of funds from one project on another project), and peer review (e.g., providing an overly positive or negative recommendation) [17]. Our results align with those of empirical studies indicating that the use of several small underpowered samples represents a more efficient research strategy (in terms of simply publishing papers, irrespective of whether the findings are correct) than does the use of one large powerful sample [18]. This is presumably why most studies have low statistical power [2], which is predicted under the rational strategy for individual scientists identified by our analysis. They are also consistent with the results of models of scientific communities, which indicate that selection for high output leads to poorer methods and increasingly high false discovery rates [19].

Current incentive structures would only be appropriate if editorial and peer review practices were much more stringent regarding the sample size and statistical power, as well as the strength of statistical evidence required of studies (Fig 4C). Critically, our model indicates how altering incentive structures, by considering more of a researcher’s output (reducing ϕ) and giving less weight to strikingly novel findings (reducing γ) when making appointment and promotion decisions, would encourage a change in researcher behaviour that improves the scientific value of research. Such a change would mean that parameters reflecting current editorial and peer review practices actually do optimise the scientific value of research (Fig 5). Effecting this change would require action by research funders and institutions. Alternatively, journals and journals editors may strive to increase the stringency of the editorial and peer review process, for example, by requiring larger sample sizes (i.e., larger *m*, generating higher statistical power) and greater statistical stringency (i.e., smaller α, increasing the proportion of significant results that are correct). Similar changes have been suggested previously [18], and some research fields have successfully implemented cultural changes—in genomics, the use of highly stringent α levels and large sample sizes is now standard practice (in part as a result of the multiple testing burden associated with genome-wide association studies) [20], while particle physics has adopted a “5 sigma” rule for declaring discovery [21]. However, our analysis is the first to show the likely impact of these strategies on the scientific value of research and how they may be complemented by top-down change to current incentive structures initiated by funders and institutions. Our model predicts that the changes to incentive structures will increase the amount of confirmatory work dramatically, but the increase in the statistical power of exploratory studies would be small (peak in Fig 1B). However, we note that the “landscape” would become less steep around the optimum (cf. Fig 1B and Fig 1A), especially in the direction of higher power (left–right). Therefore, such changes may create conditions in which it is easier to nudge researchers to do higher (> 80%) powered studies, because the (potential) cost to their individual fitness would be small.

**Fig 5:**
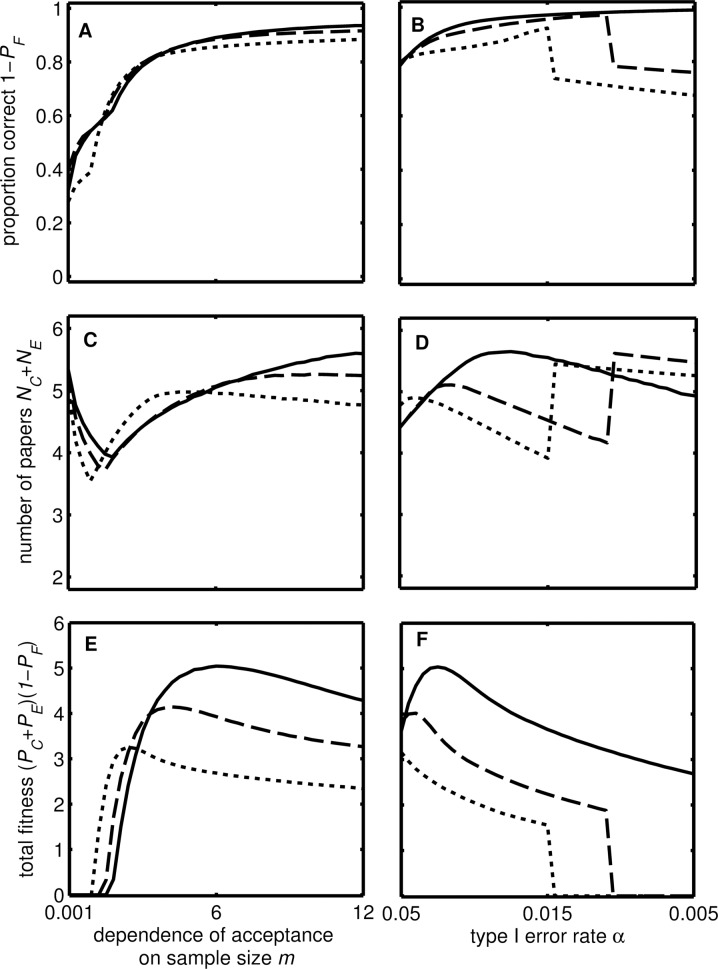
Effect of editorial stringency on total scientific output for ideal incentive structures. The figure shows the proportion of published studies that are correct 1 − *P*_*T*_ (A, B), the total number of published studies *N*_*C*_ + *N*_*C*_ (C, D), and the total scientific value of research *V*_*S*_ (E, F). We varied the following parameters: the probability of a Type I error α (A, C, E), and the dependence of acceptance on sample size *m* (B, D, F). The lines show predictions for two values of the probability that an effect is real (*f*_*E*_) and two values of the effect size (solid: *f*_*E*_ = 0.2, *r*_*C*_ *= r*_*E*_ = 0.21; dotted: *f*_*E*_ = 0.3, *r*_*C*_ *= r*_*E*_ = 0.21; dashed: *f*_*E*_ = 0.2, *r*_*C*_ *= r*_*E*_ = 0.32). Other values: *N*_*C*_ = 120, *T* = 2,000, *k* = 20, α = 0.05, σ^2^ = 1, *m* = 3, ϕ = 0.55, and γ = 0.055.

Perversely, current incentive structures may promote low-quality science, because the research strategy they encourage is more likely to produce striking (but erroneous) findings—publications from institutions that performed well in a recent research evaluation exercise report fewer measures of study quality (e.g., experimenter blinding, randomisation, etc.) than studies selected randomly from the wider literature [22]. Competition for funding and prestige may contribute to strategic-game playing [23], and it is plausible that this competition may be most pronounced at the most prestigious institutions. Current incentives that encourage scientists to build momentum around a single research focus may also be problematic, if they discourage scientists from abandoning an existing research focus (for example, because initial findings fail to replicate) and switching to a potentially more fruitful research area [24]. It is important to note that we will never attain a situation in which 100% of findings are true—indeed, this would be undesirable, as it would require us to only pursue questions with very high prestudy odds and invest considerable resources into each study to achieve near-100% statistical power. This would obviously be at the expense of novelty and discovery. Some balance is necessary. Understanding the ecosystem that gives rise to behaviours that undermine the scientific value of research is the first step towards addressing them and developing a system that strikes the optimal balance between exploratory and confirmatory research.

## Supporting Information

S1 Fig Effect of the value of *m* (shown on lines) on the function *A*. Larger values of *m* imply that larger sample sizes are required for publication in journals.
(TIF)

S2 Fig Assumptions about individual researcher fitness *V*_*R*_. Individual researcher fitness *V*_*R*_ (orange = high, black = low) as a function of the number of exploratory and confirmatory papers published for 3 values of γ (rows) and 3 values of ϕ (columns). The values capture a range that we consider reasonable. When ϕ = 0, for example, there is no diminishing return on additional papers, whereas when ϕ = 1 a single paper is valued equally to 1,000 papers. When γ = 0.0, novel findings are given equal weight to confirmatory findings, whereas when γ = 0.1 only novel findings are worth publishing because they are weighted so much more than confirmatory papers. If both are small (bottom-left) then the effect of number of both papers is linear. If γ is large and ϕ is small (top-left) then fitness is almost completely determined by the number of exploratory. If γ is small and ϕ is large (bottom-right) then fitness asymptotes at a small total number of each. If both are large (top-right) then fitness depends on both but only gets very high at a large number of exploratory.
(TIF)

S3 Fig The various possible scientific value *V*_*S*_ explored. To show the value equation we assume that individual researchers publish four studies and a proportion of them are exploratory (shown on x-axis). The value of the science may also depend on the proportion of studies that are wrong *P*_*F*_ (shown on lines). In the text we assume that the total value of science follows the equation shown in panel A, but other functions are possible. In the sensitivity analysis (S5 Fig, S6 Fig, S7 Fig, S8 Fig, S9 Fig) we show that our results are not qualitatively altered by a different choice of function.
(TIF)

S4 Fig Mechanism of the low power of exploratory studies. The optimal sample size is low because false positives can be published as exploratory studies. (A) The power of studies (blue dashed line); the number of studies that are carried out (green dotted line); the number of published articles if all studies found significant results (red solid line). (B) The number of true positives (blue dashed line); false positives (green dotted line); total articles (red solid line). The optimal sample size is at the peak total number of articles. (C) The proportion of studies that are false (blue dashed line); published (green solid line). If false positive results did not count towards researcher value, the optimal sample size would quadruple.
(TIF)

S5 Fig Sensitivity analysis for parameter values. All panels show the total value of science *V*_*S*_ [*V*_*S*_ = (*N*_*C*_ + *N*_*E*_)(1 − *P*_*F*_)] given the optimal strategy of researchers, for four values of γ and ϕ shown in the legend. The x-axes show different variables. In almost all the ranges of all parameters, a reduction in either ϕ or γ would improve *V*_*S*_, and reducing both gives the highest *V*_*S*_.
(TIF)

S6 Fig Sensitivity analysis for different scientific value function. As S5 Fig but for *V*_*S*_ = *N*_*C*_*N*_*E*_; conclusions are unchanged.
(TIF)

S7 FigSensitivity analysis for different scientific value function.As S5 Fig but for $V_{S}=(1-P_{F})\left(\frac{N_{C}N_{E}}{3}+N_{E}\right)$; conclusions are unchanged.(TIF)

S8 FigSensitivity analysis for different scientific value function.As S5 Fig but for $V_{S}=(1-P_{F})\left(\frac{N_{C}N_{E}}{3}+N_{C}\right)$; conclusions are unchanged.(TIF)

S9 FigSensitivity analysis for different scientific value function.As S5 Fig but for $V_{S}=(1-P_{F})\left(\frac{N_{C}N_{E}}{3}+\frac{N_{C}+N_{E}}{2}\right)$; conclusions are unchanged.(TIF)

S1 Table Parameters in the model and their default values. (DOCX)

S1 Text Methods. (DOCX)

S2 Text Matlab Code. (DOCX)

## Abbreviations

PPV: positive predictive value
REF: Research Excellence Framework
