# Supplementary figures and images for: Current Incentives for Scientists Lead to Underpowered Studies with Erroneous Conclusions
Source: PLoS Biol. 2016 Nov 10;14(11):e2000995. doi: 10.1371/journal.pbio.2000995 (PMC5104444; doi:10.1371/journal.pbio.2000995)

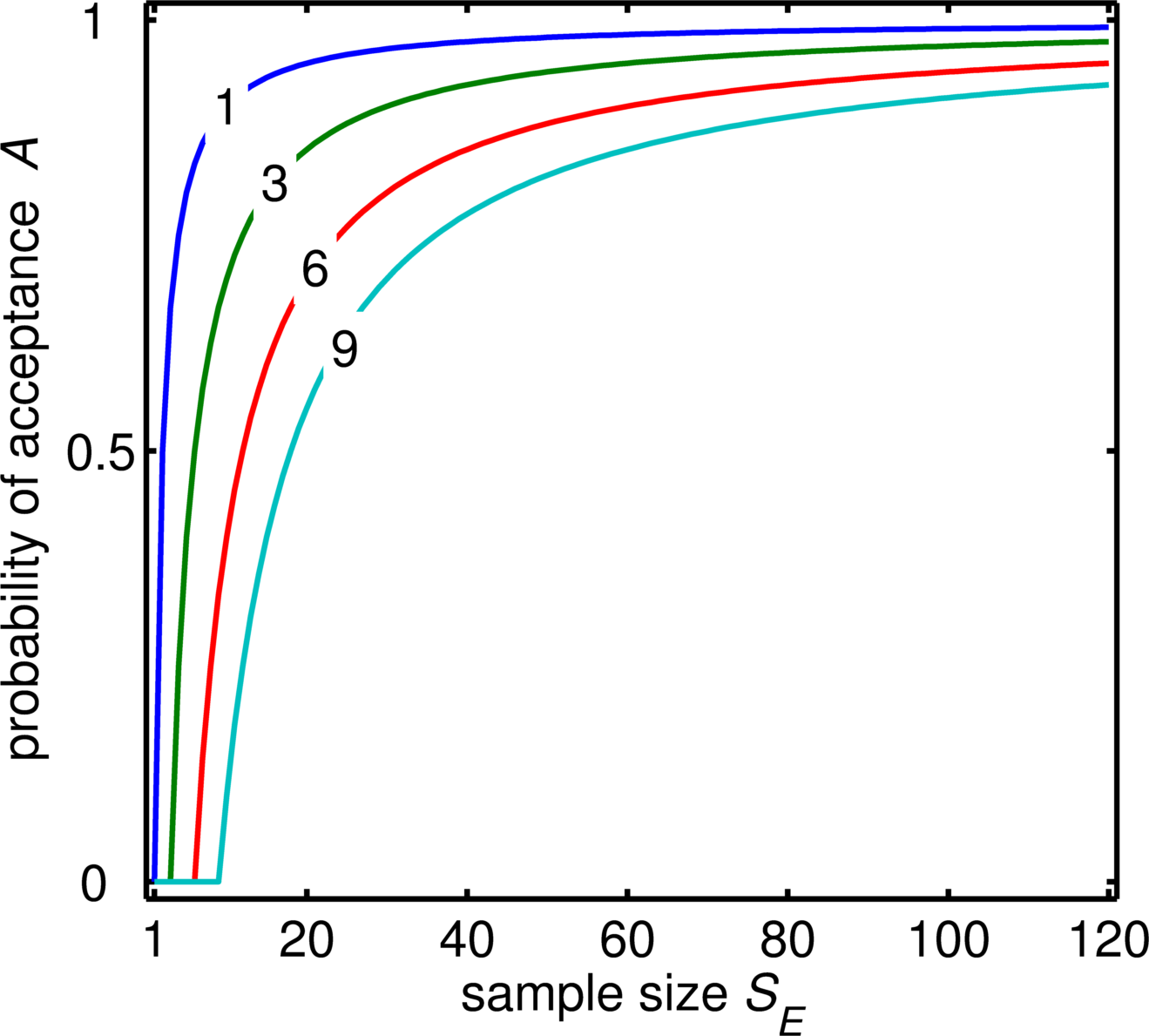

Supplement: S1 Fig — Larger values of m imply that larger sample sizes are required for publication in journals. (TIF) [file pbio.2000995.s001.tif]

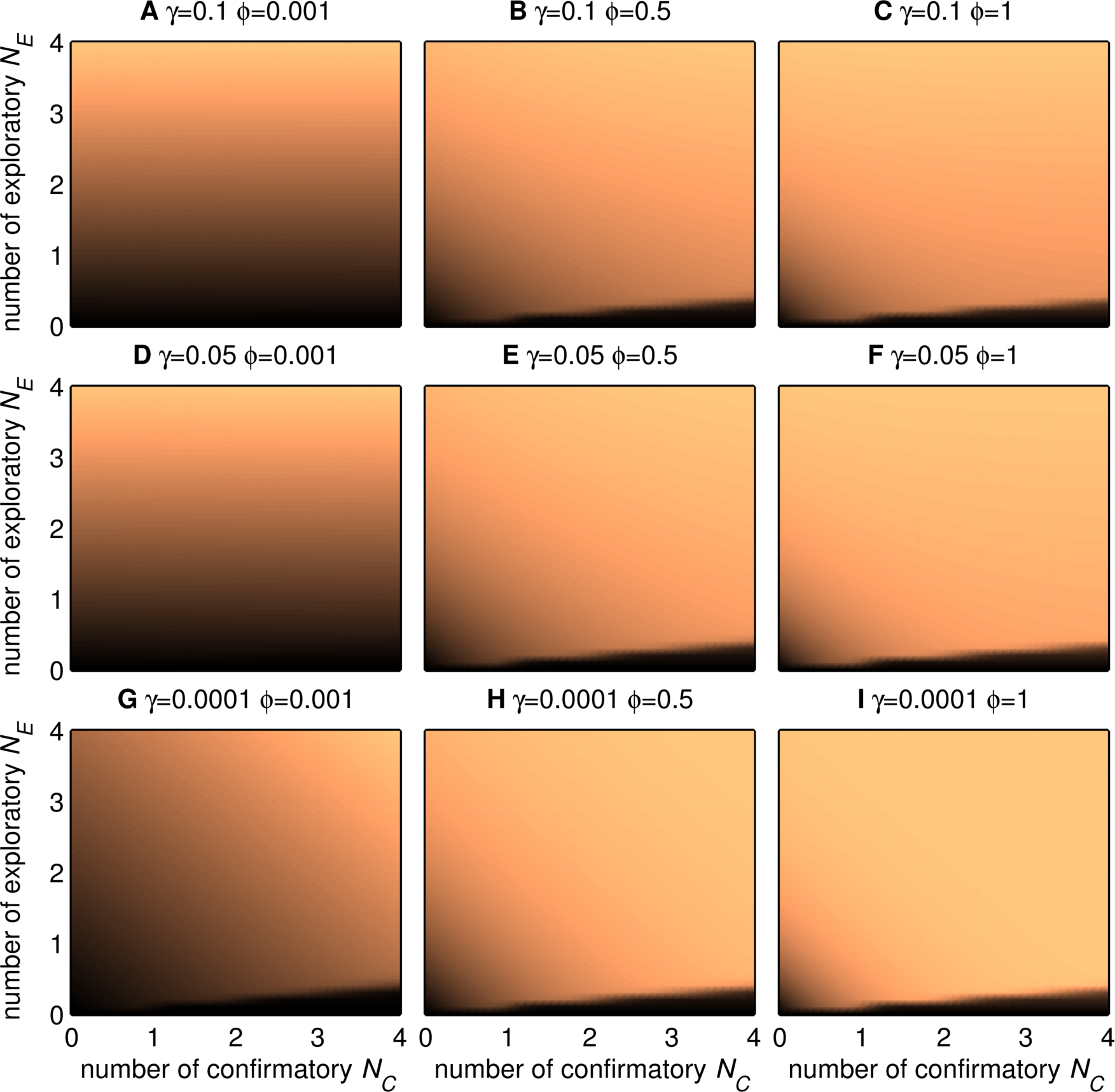

Supplement: S2 Fig — Individual researcher fitness VR (orange = high, black = low) as a function of the number of exploratory and confirmatory papers published for 3 values of γ (rows) and 3 values of ϕ (columns). The values capture a range that we consider reasonable. When ϕ = 0, for example, there is no diminishing return on additional papers, whereas when ϕ = 1 a single paper is valued equally to 1,000 papers. When γ = 0.0, novel findings are given equal weight to confirmatory findings, whereas when γ = 0.1 only novel findings are worth publishing because they are weighted so much more than confirmatory papers. If both are small (bottom-left) then the effect of number of both papers is linear. If γ is large and ϕ is small (top-left) then fitness is almost completely determined by the number of exploratory. If γ is small and ϕ is large (bottom-right) then fitness asymptotes at a small total number of each. If both are large (top-right) then fitness depends on both but only gets very high at a large number of exploratory. (TIF) [file pbio.2000995.s002.tif]

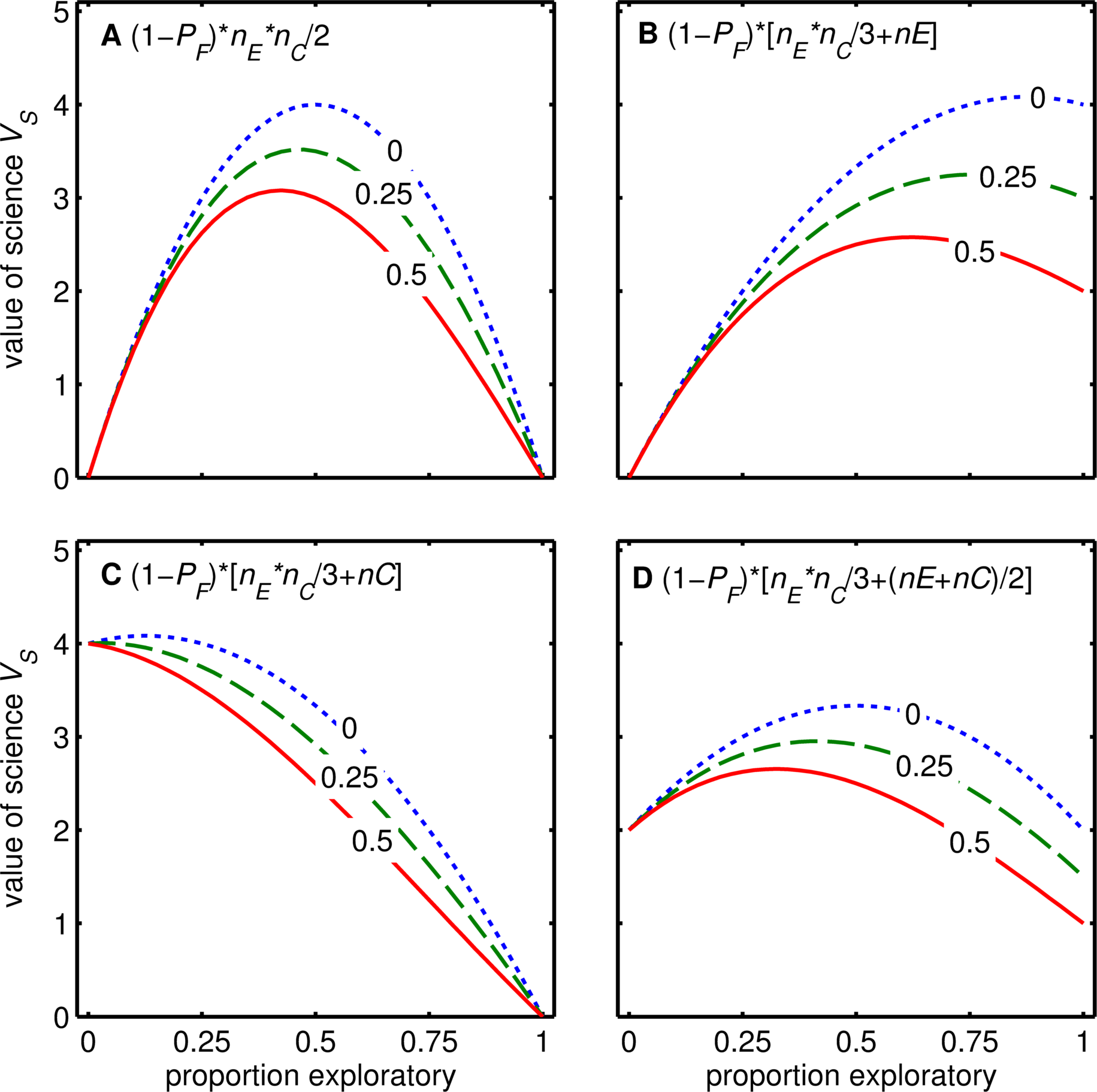

Supplement: S3 Fig — To show the value equation we assume that individual researchers publish four studies and a proportion of them are exploratory (shown on x-axis). The value of the science may also depend on the proportion of studies that are wrong PF (shown on lines). In the text we assume that the total value of science follows the equation shown in panel A, but other functions are possible. In the sensitivity analysis (S5 Fig, S6 Fig, S7 Fig, S8 Fig, S9 Fig) we show that our results are not qualitatively altered by a different choice of function. (TIF) [file pbio.2000995.s003.tif]

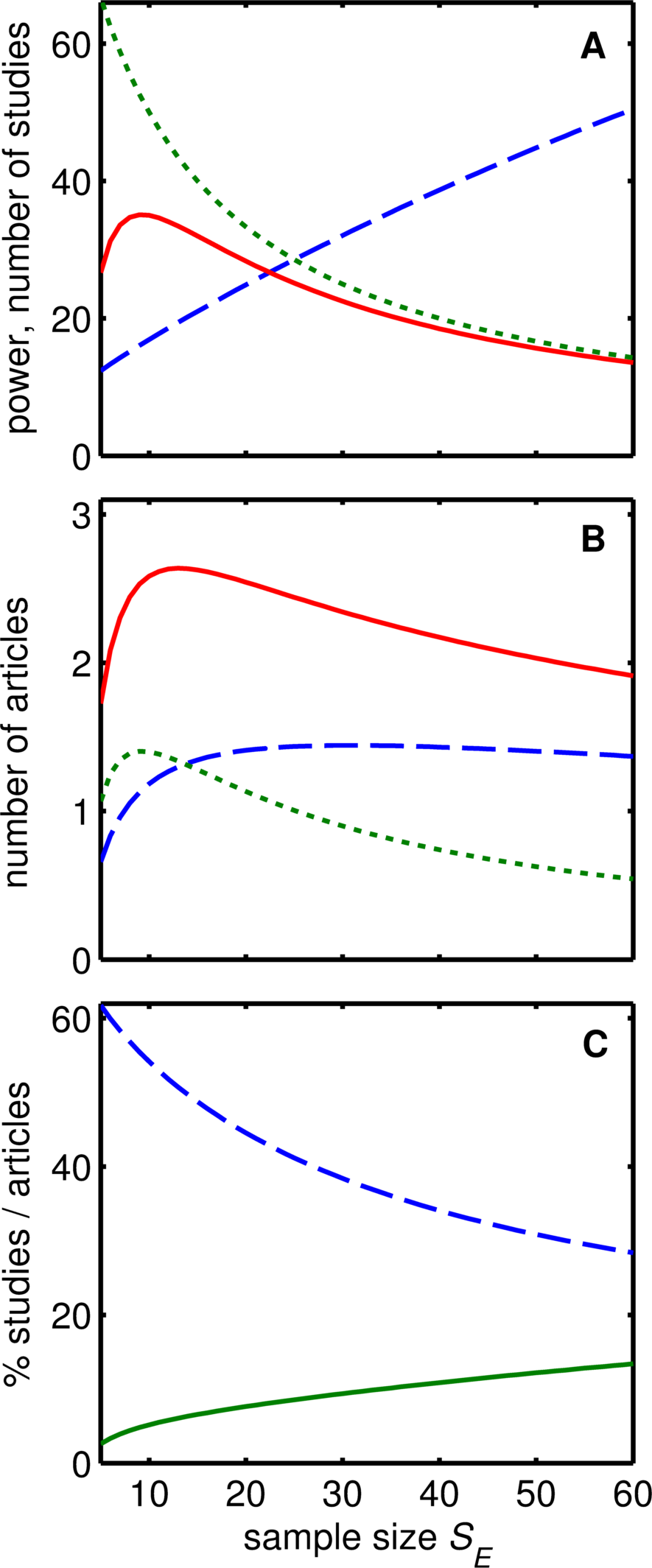

Supplement: S4 Fig — The optimal sample size is low because false positives can be published as exploratory studies. (A) The power of studies (blue dashed line); the number of studies that are carried out (green dotted line); the number of published articles if all studies found significant results (red solid line). (B) The number of true positives (blue dashed line); false positives (green dotted line); total articles (red solid line). The optimal sample size is at the peak total number of articles. (C) The proportion of studies that are false (blue dashed line); published (green solid line). If false positive results did not count towards researcher value, the optimal sample size would quadruple. (TIF) [file pbio.2000995.s004.tif]

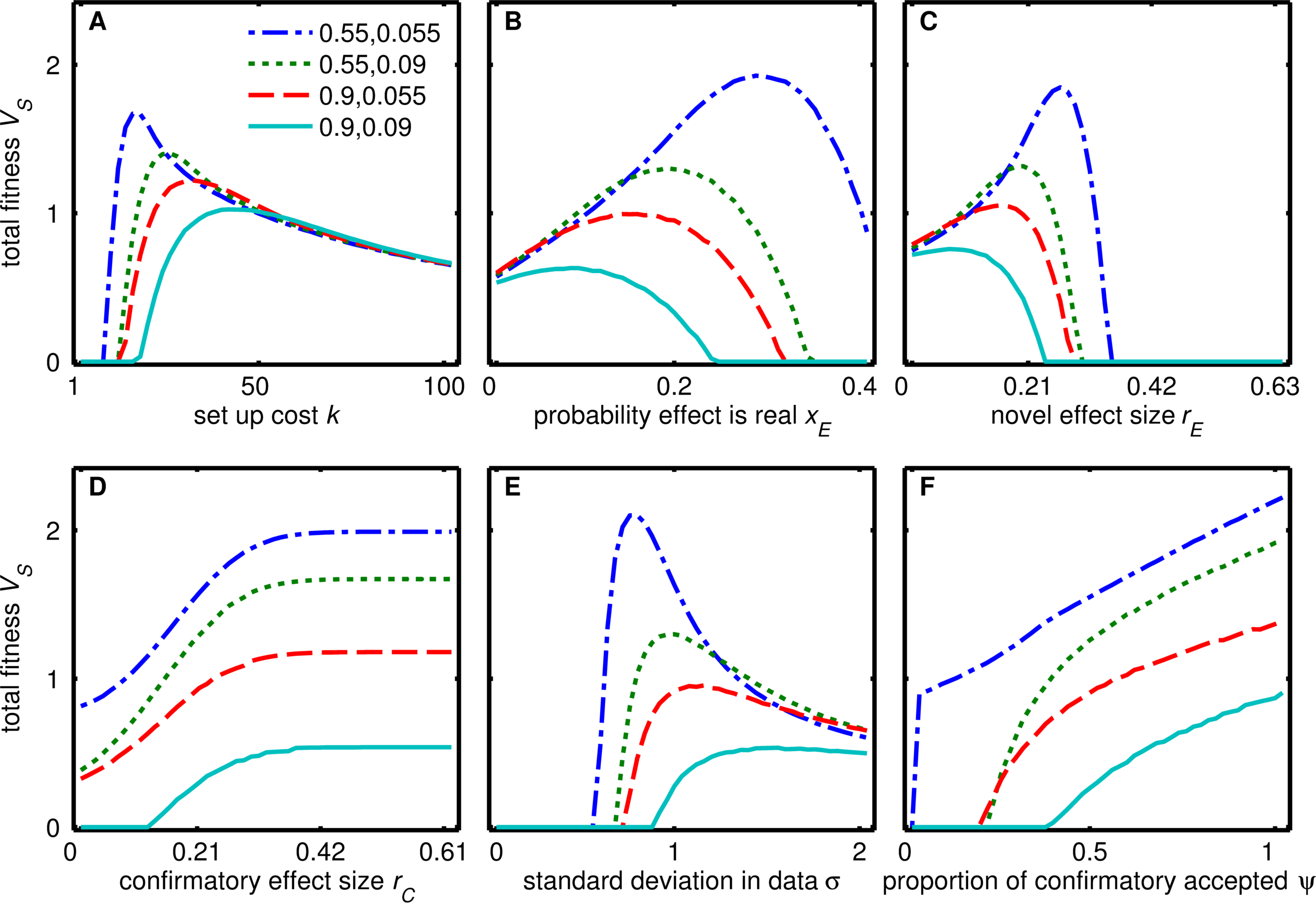

Supplement: S5 Fig — All panels show the total value of science VS [VS = (NC + NE)(1 − PF)] given the optimal strategy of researchers, for four values of γ and ϕ shown in the legend. The x-axes show different variables. In almost all the ranges of all parameters, a reduction in either ϕ or γ would improve VS, and reducing both gives the highest VS. (TIF) [file pbio.2000995.s005.tif]

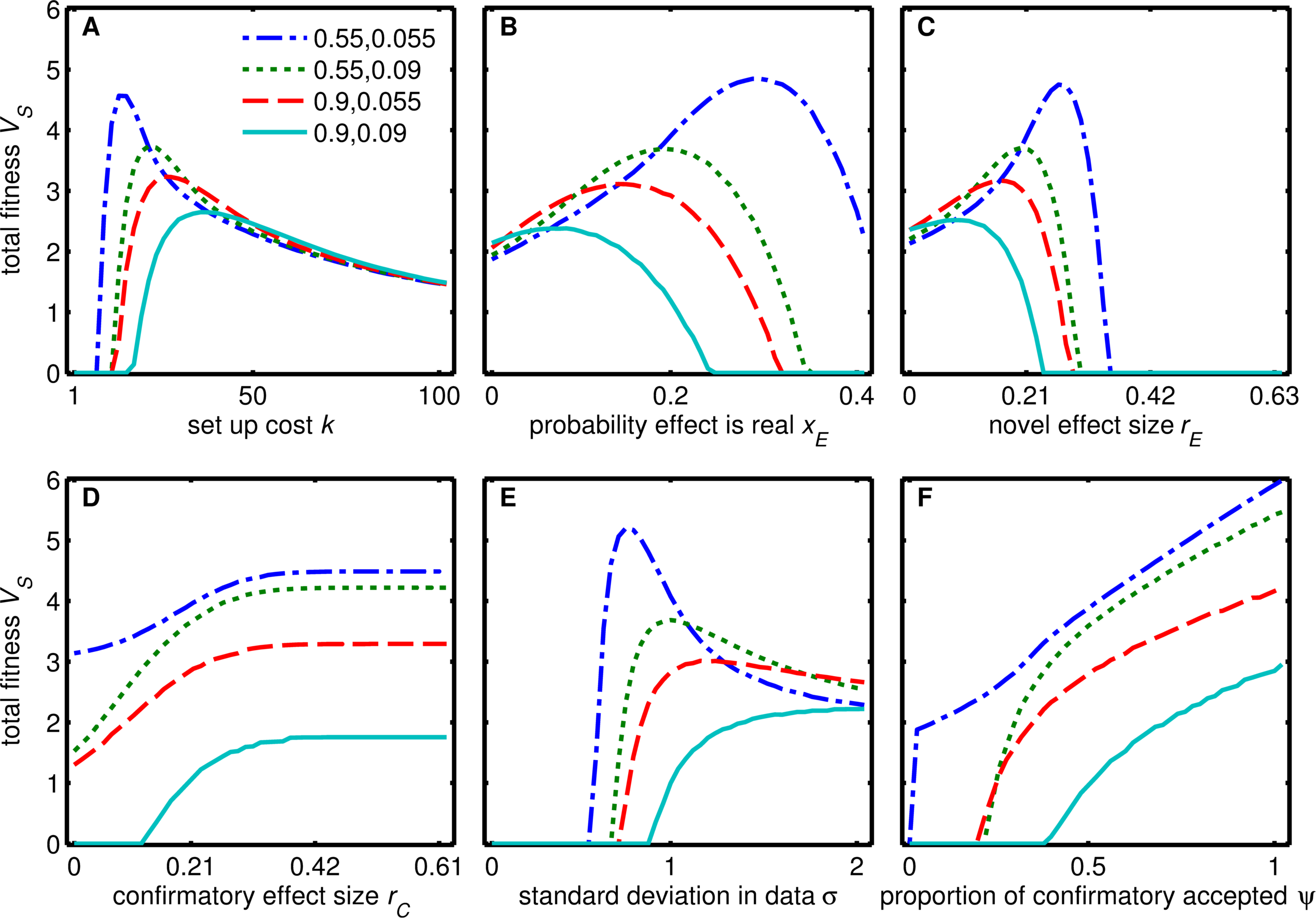

Supplement: S6 Fig — As S5 Fig but for VS = NCNE; conclusions are unchanged. (TIF) [file pbio.2000995.s006.tif]

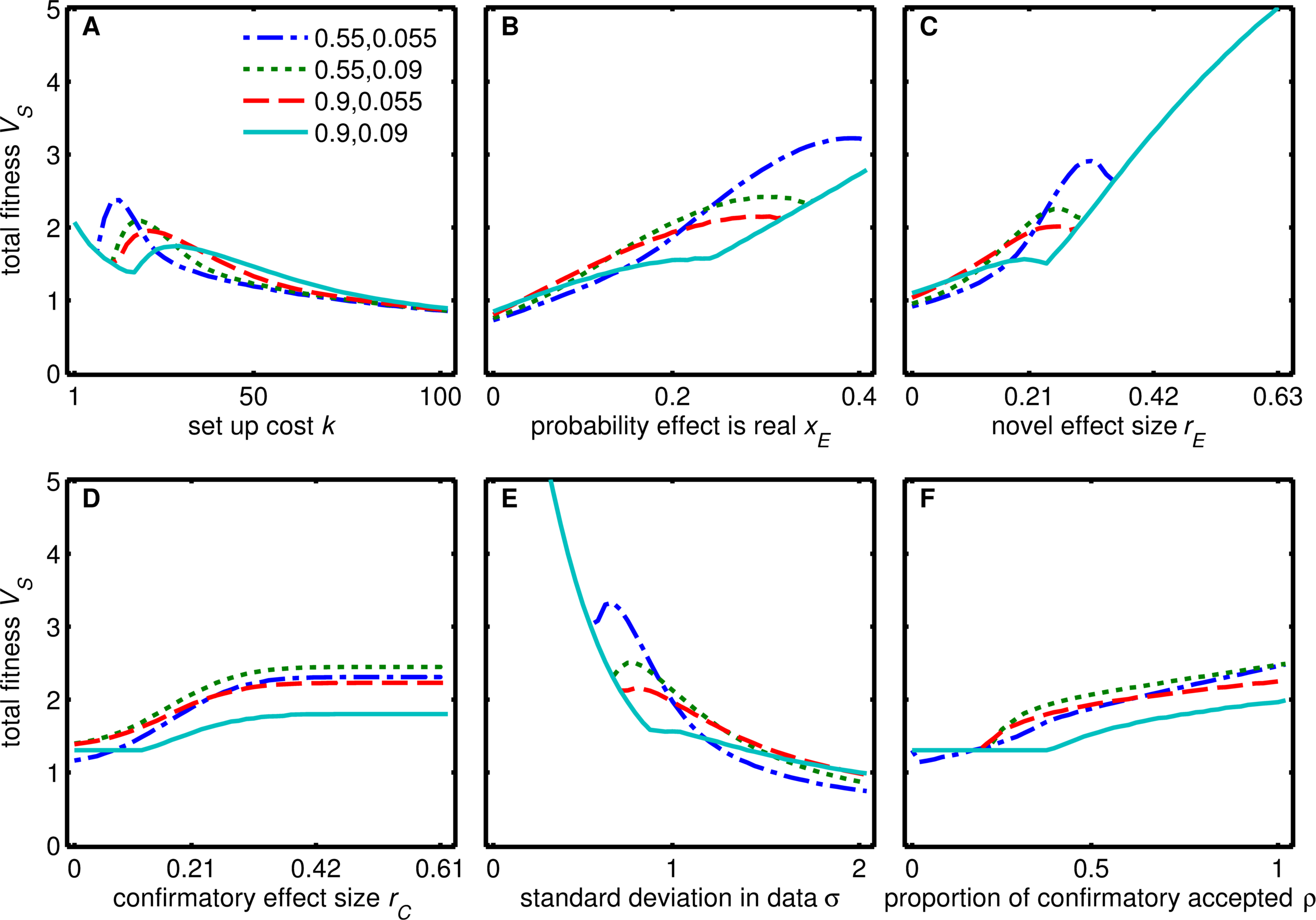

Supplement: S7 Fig — As S5 Fig but for VS=(1−PF)(NCNE3+NE); conclusions are unchanged. (TIF) [file pbio.2000995.s007.tif]

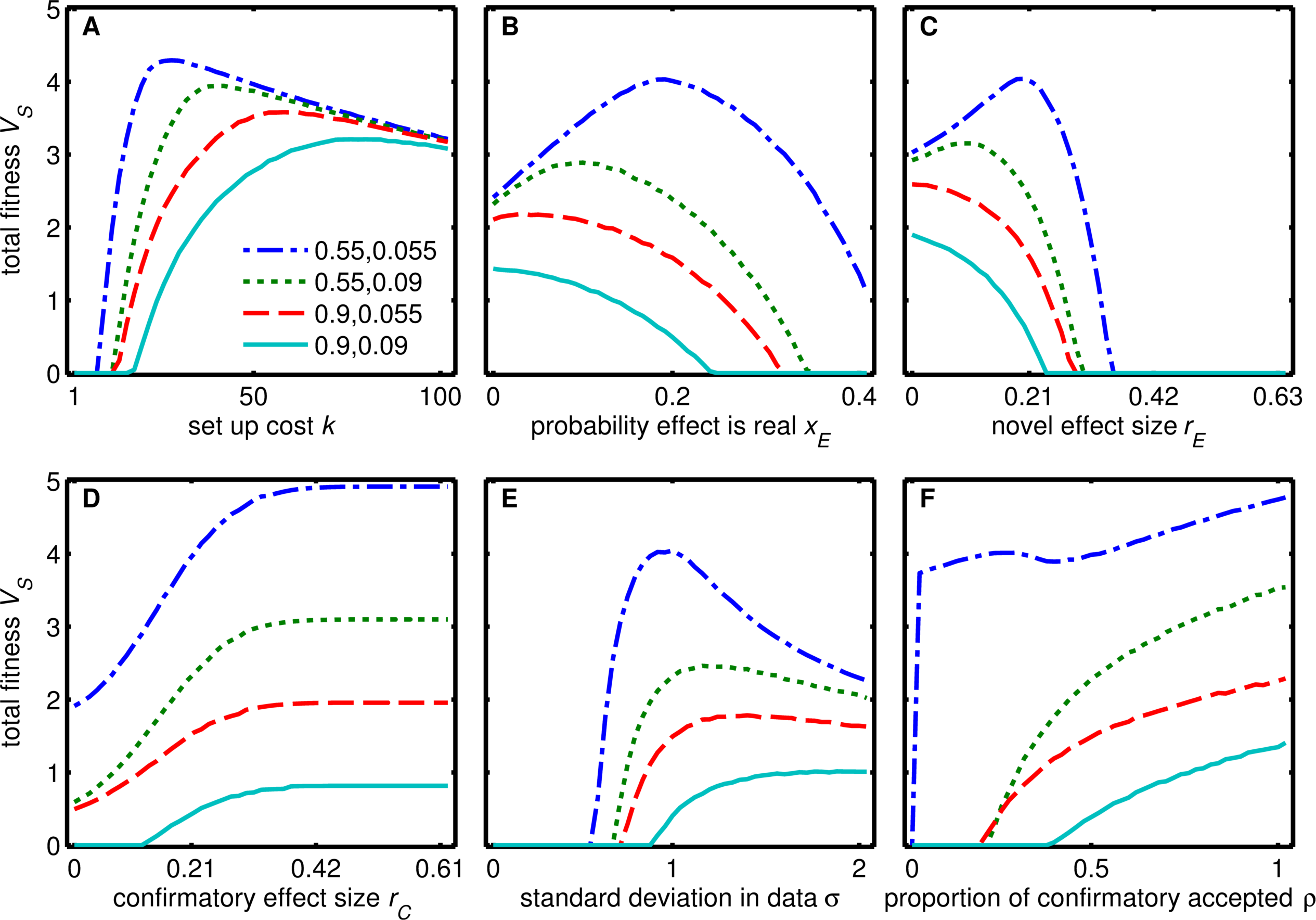

Supplement: S8 Fig — As S5 Fig but for VS=(1−PF)(NCNE3+NC); conclusions are unchanged. (TIF) [file pbio.2000995.s008.tif]

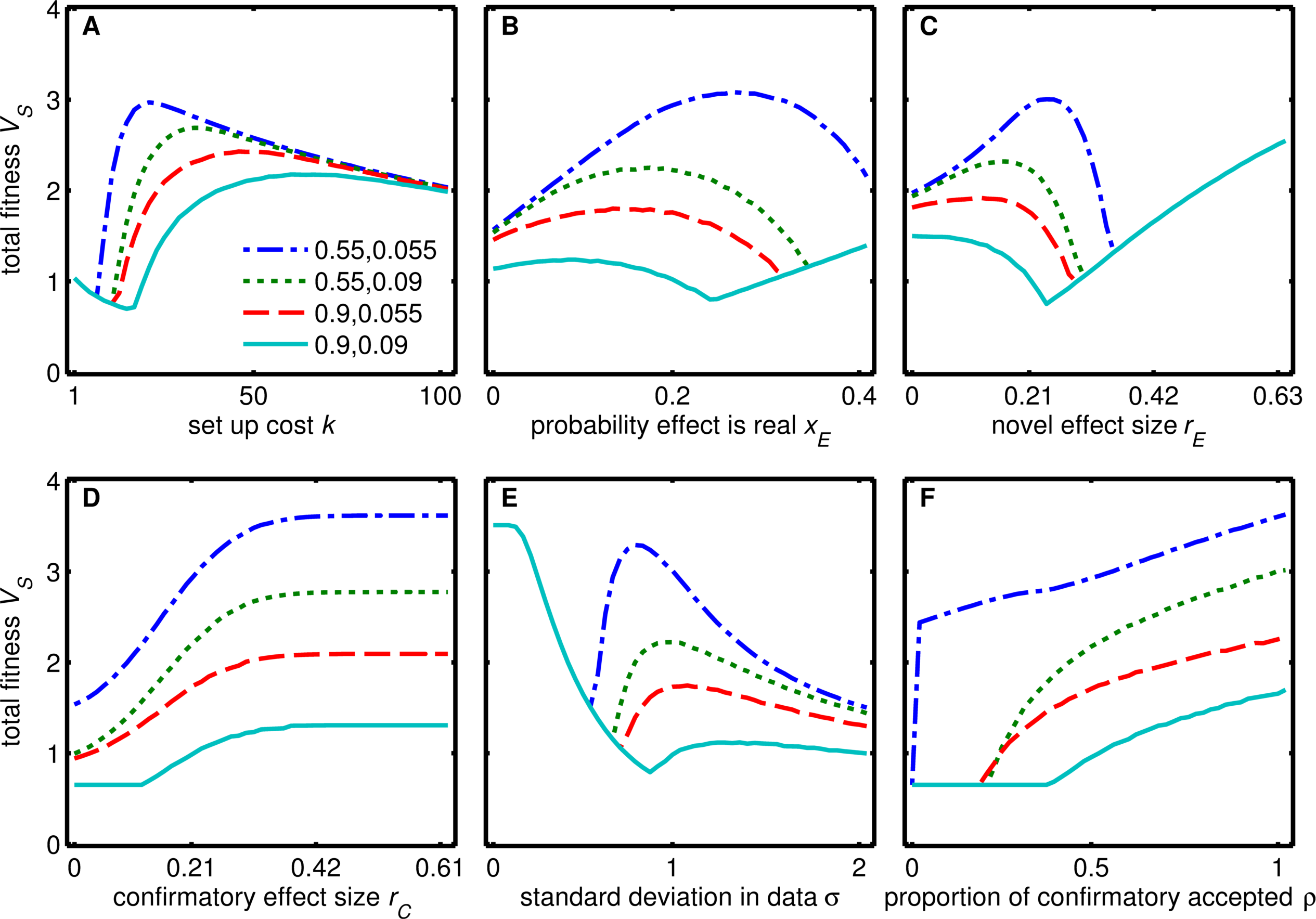

Supplement: S9 Fig — As S5 Fig but for VS=(1−PF)(NCNE3+NC+NE2); conclusions are unchanged. (TIF) [file pbio.2000995.s009.tif]
