# Supplementary material for: Current Incentives for Scientists Lead to Underpowered Studies with Erroneous Conclusions
Source: PLoS Biol. 2016 Nov 10;14(11):e2000995. doi: 10.1371/journal.pbio.2000995 (PMC5104444; doi:10.1371/journal.pbio.2000995)
Supplement: S1 Table — (DOCX) [file pbio.2000995.s010.docx]

| **Parameter** | **Symbol** | **Values** |
| --- | --- | --- |
| ***Controlled by scientists*** |  |  |
| Proportion of effort on exploratory research | *θ* | – |
| Sample size of exploratory studies | *S_E_* | – |
| Sample size of confirmatory studies | *S_C_* | 120 |
| Number of published exploratory studies | *N_E_* | – |
| Number of published confirmatory studies | *N_C_* | – |
| Individual value of researchers | *V_R_* | – |
| ***Ecosystem parameters*** |  |  |
| Total resource available | *T* | 2000 |
| Set-up cost of study | *k* | 20 |
| Probability that an exploratory study is looking at a real effect | *f_E_* | 0.2, 0.3 |
| Effect size in exploratory studies | *r_E_* | 0.21, 0.32 |
| Effect size in confirmatory studies | *r_C_* | 0.21, 0.32 |
| Population standard deviation | *σ^2^* | 1 |
| Type I error rate | *α* | 0.05 |
| Probability that non-significant confirmatory study is published | *ψ* | 0.5 |
| Sample size stringency | *m* | 3, 6 |
| Maximum ratio of confirmatory to exploratory studies | *ρ* | 10 |
| Diminishing returns of publishing | *ϕ* | 0.55, 0.9 |
| Bonus for published exploratory studies | *γ* | 0.055, 0.09 |
| Total value of the science | *V_S_* | – |
