# Supplementary material for: Current Incentives for Scientists Lead to Underpowered Studies with Erroneous Conclusions
Source: PLoS Biol. 2016 Nov 10;14(11):e2000995. doi: 10.1371/journal.pbio.2000995 (PMC5104444; doi:10.1371/journal.pbio.2000995)
Supplement: S1 Text — (DOCX) [file pbio.2000995.s011.docx]

We consider a hypothetical rational researcher aiming to maximise their chances of career progression. We assume that the researcher optimises the payoff that results from dividing a fixed research budget (e.g. time or money) into (i) exploratory studies that may identify novel phenomena, and (ii) confirmatory studies that attempt to replicate previous findings, and deciding how much to invest in each study. Specifically, the researcher makes three decisions: 1) the proportion of total sampling to spend on exploratory studies (denoted by *θ*); 2) the sample size of each exploratory study (*S_E_*); and 3) the sample size of each confirmatory study (*S_C_*).

For simplicity we assume that there is a typical effect size for the phenomena investigated within a particular field, but we allow this average to differ between exploratory and confirmatory studies. The sample size gives an estimate of the statistical power of each study, which in turn controls the probabilities of Type I and Type II errors and so the probability of observing an effect (either a true positive or a false positive), and of acceptance by a journal. The high statistical power of confirmatory studies means that the results are informative regardless of statistical significance so we assume that all are published with fixed probability ψ, but that exploratory studies are only published if they get a statistically significant result.

Statistical power is a function of the probability density function of the Gaussian distribution, and so is difficult to work with, but an acceptable approximation of the dependence of the statistical power *W_i_* of study type *i* () with sample size *S_i_* is given by:

$W_{i}=\frac{1}{2}-\frac{1}{2}\frac{H_{i}-r_{i}^{2}}{\sqrt{(H_{i}-r_{i}^{2})^{2}+\delta}}\sqrt{1-e^{-\frac{2S_{i}}{\pi\sigma^{2}}\left(H_{i}-r_{i}^{2}\right)}}$ (S1)

where

.

In equation (A1) σ^2^ is the population variance, *r_i_* is the effect size for exploratory or confirmatory studies, α is the Type I error rate (i.e., the critical *P* value within a null hypothesis significance testing framework), and δ is a small number (10^-12^) used in the approximation of sign(*H–r*^2^).

We assume that the likelihood of acceptance by a journal editor is influenced by sample size *per se*, before consideration of the effects themselves. A reasonable function that captures the idea that the probability of acceptance on the basis of sample size alone is small when *S_i_* is very small and tends to unity otherwise as *S_i_* becomes large is:

$A=1-\frac{m}{S_{i}}$ (S2)

where *m* is a positive constant. As *m* increases, journal editors demand larger sample sizes before accepting studies for publication (S1 Fig). This equation tends to minus infinity at small *S_i_*, but this region is never optimal so this does not affect predictions.

The number of publications from exploratory studies (*N_E_*) depends on two probabilities: 1) the probability of correctly identifying a novel phenomenon (i.e., a true positive), which is the product of the statistical power and the probability that an exploratory study is indeed investigating an effect that is real (*f_E_*); and 2) the probability of finding a statistically significant result when the phenomenon does not exist (i.e., a false positive), which is the product of the Type I error rate (α) and the probability that an exploratory study is in fact investigating an effect that is *not* real (1–*f_E_*). We assume that all non-significant results from exploratory studies are not published (i.e., the file drawer phenomenon). *N_E_* is the product of the number of exploratory studies conducted, the probability of acceptance given the sample size, and the probability of getting a statistically significant result:

$N_{E}=\frac{\theta T}{k+2S_{E}}A\left[W_{E}f_{E}+\alpha(1-f_{E})\right]$, (S3)

where *T* is the total number of samples that can be collected (i.e., total resources) and *k* is the set-up cost for any study. The first term in the squared brackets is the probability of a true positive result, while the second is the probability of a false positive result.

The proportion of published exploratory studies that are false is:

$P_{F,E}=\frac{\alpha(1-f_{E})}{W_{E}f_{E}+\alpha(1-f_{E})}$. (S4)

Since confirmatory studies will build on the findings of exploratory studies, the probability that a confirmatory study is looking at a real effect (*f_C_*) is equal to the probability that a published exploratory study is correct (*f_C_*=1-*P_F,E_*). The number of confirmatory studies that are published (*N_C_*) is the sampling effort put into all confirmatory studies divided by the sampling effort of each study multiplied by the probability they are accepted:

$N_{C}=\frac{(1-\theta)T}{k+2S_{C}}\left[\left(1-P_{F,E}\right)W_{C}+P_{F,E}\alpha+\psi P_{F,E}(1-\alpha)+\psi\left(1-P_{F,E}\right)\left(1-W_{C}\right)\right]$,(S5)

The terms in the squared brackets are respectively, the probability of true positive, false positive, true negative, false negative.

The probability of false negatives will therefore be $(1-W_{E})f_{E}$ for exploratory studies and $(1-W_{C})f_{C}$for confirmatory studies, so the total probability any study commits a false negative is:

$P_{FN}=\frac{(1-\theta)T}{k+2S_{C}}(1-W_{C})f_{C}+\frac{\theta T}{k+2S_{E}}(1-W_{E})f_{E}$. (S6)

The proportion of published studies that are false (false positive or false negative) is:

$P_{F}=\frac{\frac{\theta T}{k+2S_{E}}A\left[\alpha(1-f_{E})\right]+\frac{(1-\theta)T}{k+2S_{C}}\left[P_{F,E}\alpha+\psi\left(1-P_{F,E}\right)\left(1-W_{C}\right)\right]}{N_{E}+N_{C}}$. (S7)

The total fitness of the researcher is assumed to depend on total number of publications (with diminishing returns) with a weighting for exploratory studies. We assume that the number of confirmatory studies per exploratory study is limited to ρ (ρ=10), by calculating the number of *valuable* confirmatory studies as:

$\hat{N}_{C}=\frac{N_{C}}{1+e^{\frac{N_{C}}{N_{E}+\varepsilon}-\rho}}$ (S8)

where ε is a small number (10^-6^) that controls the steepness of the slope. Equation (A8) implies that the value of confirmatory studies is around unity when *N_C_<N_E_* and around zero when *N_C_>N_E_*.

The value of an individual researcher is given by:

, (S9)

In equation A9 ϕ controls how quickly the value of the total number of publications diminishes, and γ controls the extra weighting given to published exploratory studies. The dependence of equation (A9) on the number of published exploratory and confirmatory studies is shown in S2 Fig for representative values of γ and ϕ.

We simplify our analysis by assuming a fixed value for *S_C_* and finding a strategy in only two-dimensional (θ, *S_E_*) space. We assume *S_C_* = 120 so that the statistical power of confirmatory studies is *W_C_* ≈ 0.80 (and where *A* is almost unity) at the values of other parameters.

We are especially interested in how the optimal strategy changes with the weight given to novel findings γ and how quickly the value of the total number of publications diminishes ϕ. We can find the optimal strategy (*θ**,*S_E_**): the combination of proportion of effort into exploratory studies and the sample size of exploratory studies that maximises *V_S_*. See Supplementary Information for the Matlab code.
