# Supplementary material for: Current Incentives for Scientists Lead to Underpowered Studies with Erroneous Conclusions
Source: PLoS Biol. 2016 Nov 10;14(11):e2000995. doi: 10.1371/journal.pbio.2000995 (PMC5104444; doi:10.1371/journal.pbio.2000995)
Supplement: S2 Text — (DOCX) [file pbio.2000995.s012.docx]

% PUB_STRAT_CLEAN solves the simultaneous differential equations model of Higginson & Munafo.

% This code carries out the sensitivity analysis of the appendix.

% The remaining code is available at: https://zenodo.org/record/155251#.V-jQSDKZMi4

% numeric parameters

V_expWeighting=0.09; % funding bias to sig exploratory

V_maxSample=2000; % maximum sample size

V_effectRealExp=0.2; % probability exploratory has real effect

V_effectSizeExp=0.21; % effect size of exploratory

V_effectSizeCon=0.21; % effect size of confirmatory

V_falsepos=0.05; % alpha

V_stdev=1; % standard deviation

V_setupcost=20; % baseline cost of each experiment

V_minsamp=3; % minimum community acknowledged sample size

V_sampsizeCon=100; % size of confirmatory test

V_propAcceptCon=0.5; % proportion of confirmatory accepted

V_dimintot=0.9; % rate of diminshing value of publications

V_maxratio=10; % maximum power allowed

%[~,~,panlabels]=subplotarrange(6);

% create symbolic things

syms sampsizeExp propExp expWeighting maxSample effectRealExp maxratio

syms effectSizeExp effectSizeCon falsepos stdev setupcost minsamp sampsizeCon propAcceptCon dimintot

%%%%%%%% MODEL %%%%%%%%%%%%%%%%%%%%%%%%%%%%%%%%%%%%%%%%%%%%%%%%%%%%%%%%%%%%

% approximation for power

mstar=(-(pi()*stdev^2./(2*sampsizeExp)).*log(4*falsepos.*(1-falsepos)));

brac=(2./(pi()*(stdev^2./sampsizeExp))).*(sqrt(mstar)-effectSizeExp).^2;

signm=((mstar-effectSizeExp^2))./(sqrt((mstar-effectSizeExp^2).^2)+10^-12);

powerExp=0.5-0.5.*signm.*sqrt(1-exp(-1*brac));

% approximation for power

mstar=(-(pi()*stdev^2./(2*sampsizeCon)).*log(4*falsepos.*(1-falsepos)));

brac=(2./(pi()*(stdev^2./sampsizeCon))).*(sqrt(mstar)-effectSizeCon).^2;

signm=((mstar-effectSizeCon^2))./(sqrt((mstar-effectSizeCon^2).^2)+10^-12);

powerCon=0.5-0.5.*signm.*sqrt(1-exp(-1*brac));

% acceptability by journal

accprob=1-minsamp./sampsizeExp;

% all exploratory non-sig are file drawered

nExp=propExp.*(maxSample./(setupcost+2*sampsizeExp)).*accprob.*(powerExp.*effectRealExp+falsepos.*(1-effectRealExp));

% number of exploratory wrong

nWrongExp=propExp.*(maxSample./(setupcost+2*sampsizeExp)).*accprob.*falsepos.*(1-effectRealExp);

% publication in exploratory and confirmatory

% proportion of confirmatory are published if not significant

% all published if significant

pWrongExp=(nWrongExp+10.^-6)/(nExp+10.^-6);

nCon=(1-propExp)*maxSample./(setupcost+2*sampsizeCon)*...

((powerCon.*(1-pWrongExp)+falsepos.*pWrongExp)... % significant: TP & FP

+propAcceptCon*((1-powerCon).*(1-pWrongExp)+(1-falsepos).*pWrongExp)); % non-sig: FN & TN

% total false negatives

nFalseNeg=(1-propExp).*(maxSample./(setupcost+2*sampsizeCon)).*((1-powerCon).*(nWrongExp/nExp))...

+propExp.*(maxSample./(setupcost+2*sampsizeExp)).*((1-powerExp).*effectRealExp);

propFalseNeg=nFalseNeg./((1-propExp).*(maxSample./(setupcost+2*sampsizeCon))+propExp.*(maxSample./(setupcost+2*sampsizeExp)));

% wrong published: false positive and false negative

nWrong=(1-propExp).*(maxSample./(setupcost+2*sampsizeCon)).*propAcceptCon*(falsepos.*(1-(nWrongExp/nExp))+(1-powerCon).*(nWrongExp/nExp))...

+propExp.*(maxSample./(setupcost+2*sampsizeExp)).*accprob.*falsepos.*(1-effectRealExp);

propWrong=nWrong./(nCon+nExp);

% fitness is sum of two things - weighted impact factor and number with diminishing returns

fitness=(expWeighting.*nExp)+(1-exp(-dimintot*(nExp+nCon.*(1./(1+exp((nCon/(nExp+10^-6)-maxratio)))))));%

% SENSIVITY ANALYSIS

if dosens==1

nvarvals=51;

dmnvals=[0.55 0.55 0.9 0.9]%0.5:0.05:0.8%

eWvals=[ 0.055 0.09 0.055 0.09]%0.05:0.005:0.08%;

FitSci=nan(5,6,nvarvals,length(dmnvals));

optsval4D=nan(5,6,nvarvals,length(dmnvals));

optpval4D=nan(5,6,nvarvals,length(dmnvals));

optnCon4D=nan(5,6,nvarvals,length(dmnvals));

optnExp4D=nan(5,6,nvarvals,length(dmnvals));

optwrong4D=nan(5,6,nvarvals,length(dmnvals));

% loop over the fitness functions

for fitfuncnum=1:5

% loop over the fitness parameters

for fitpars=1:length(dmnvals)

V_expWeighting=eWvals(fitpars);

V_dimintot=dmnvals(fitpars);

% loop over the variable of interest

for varn=1:6

% check the values of the others

V_minsamp=3;

V_falsepos=0.05; % alpha

V_setupcost=20; % baseline cost of each experiment

V_effectRealExp=0.2; % probability exploratory has real effect

V_effectSizeExp=0.21; % effect size of exploratory

V_effectSizeCon=0.21; % effect size of confirmatory

V_stdev=1; % standard deviation

V_propAcceptCon=0.5; % for one of the analyses

% loop over the values

for x1=1:nvarvals

switch varn

case 1

V_setupcost=100*(x1)/(nvarvals);

case 2

V_effectRealExp=0.2*2*(x1)/(nvarvals);

case 3

V_effectSizeExp=0.21*3*(x1)/(nvarvals);

case 4

V_effectSizeCon=0.21*3*(x1)/(nvarvals);

case 5

V_stdev=2*(x1)/(nvarvals);

case 6

V_propAcceptCon=(x1-1)/(nvarvals-1);

end

paramnames={expWeighting dimintot setupcost effectRealExp effectSizeExp effectSizeCon minsamp falsepos maxSample stdev propAcceptCon sampsizeCon maxratio };

paramvals={V_expWeighting V_dimintot V_setupcost V_effectRealExp V_effectSizeExp V_effectSizeCon V_minsamp V_falsepos V_maxSample V_stdev V_propAcceptCon V_sampsizeCon V_maxratio };

% simplify things

optwrong=subs(propWrong,paramnames,paramvals);

optnCon=subs(nCon,paramnames,paramvals);

optnExp=subs(nExp,paramnames,paramvals);

V_fitness=subs(fitness,paramnames,paramvals);

% get the optimal strategy

[optx,opty]=findmaxsym(V_fitness,propExp,sampsizeExp,0,[0 1],[1 V_maxSample/20],[1 1]);

optx=optx(~isnan(optx));

opty=opty(~isnan(opty));

optpval4D(fitfuncnum,varn,x1,fitpars)=optx(numel(optx));

optsval4D(fitfuncnum,varn,x1,fitpars)=opty(numel(opty));

% fill in the other stuff

optwrong4D(fitfuncnum,varn,x1,fitpars)=double(subs(optwrong,{propExp,sampsizeExp},{optpval4D(fitfuncnum,varn,x1,fitpars),optsval4D(fitfuncnum,varn,x1,fitpars)}));

optnCon4D(fitfuncnum,varn,x1,fitpars)=double(subs(optnCon,{propExp,sampsizeExp},{optpval4D(fitfuncnum,varn,x1,fitpars),optsval4D(fitfuncnum,varn,x1,fitpars)}));

optnExp4D(fitfuncnum,varn,x1,fitpars)=double(subs(optnExp,{propExp,sampsizeExp},{optpval4D(fitfuncnum,varn,x1,fitpars),optsval4D(fitfuncnum,varn,x1,fitpars)}));

switch fitfuncnum

case 1

FitSci(fitfuncnum,varn,x1,fitpars)=(optnCon4D(fitfuncnum,varn,x1,fitpars).*optnExp4D(fitfuncnum,varn,x1,fitpars)).*(1-optwrong4D(fitfuncnum,varn,x1,fitpars))/2;

case 2

FitSci(fitfuncnum,varn,x1,fitpars)=(optnCon4D(fitfuncnum,varn,x1,fitpars).*optnExp4D(fitfuncnum,varn,x1,fitpars));

case 3

FitSci(fitfuncnum,varn,x1,fitpars)=(optnExp4D(fitfuncnum,varn,x1,fitpars)+optnCon4D(fitfuncnum,varn,x1,fitpars).*optnExp4D(fitfuncnum,varn,x1,fitpars)/3).*(1-optwrong4D(fitfuncnum,varn,x1,fitpars));

case 4

FitSci(fitfuncnum,varn,x1,fitpars)=(optnCon4D(fitfuncnum,varn,x1,fitpars)+optnCon4D(fitfuncnum,varn,x1,fitpars).*optnExp4D(fitfuncnum,varn,x1,fitpars)/3).*(1-optwrong4D(fitfuncnum,varn,x1,fitpars));

case 5

FitSci(fitfuncnum,varn,x1,fitpars)=((optnCon4D(fitfuncnum,varn,x1,fitpars)+optnExp4D(fitfuncnum,varn,x1,fitpars))/2+(optnCon4D(fitfuncnum,varn,x1,fitpars).*optnExp4D(fitfuncnum,varn,x1,fitpars))/3).*(1-optwrong4D(fitfuncnum,varn,x1,fitpars));

end

end

end

end

end

end
